# Best Practices in Implementing the VA’s Life-Sustaining Treatment Decisions Initiative

**DOI:** 10.1093/geroni/igaa057.2710

**Published:** 2020-12-16

**Authors:** Leah Haverhals, Jennifer Kononowech, Courtney Bauers, Chelsea Manheim, Cari Levy

**Affiliations:** 1 Department of Veterans Affairs, Denver, Colorado, United States; 2 VA Ann Arbor Healthcare System, Ann Arbor, Michigan, United States; 3 Denver-Seattle Center of Innovation for Veteran-Centered and Value-Driven Care, Aurora, Colorado, United States; 4 Rocky Mountain Regional Medical Center, Aurora, Colorado, United States; 5 VA Eastern Colorado Health Care System, Aurora, Colorado, United States

## Abstract

To study use and completion of Life Sustaining Treatment (LST) templates across the Department of Veterans Affairs (VA) healthcare system, we designed a qualitative study to interview VA sites we identified who had high rates of LST template completion between July 1, 2018 (the official implementation start date) and March 2019. We then conducted site visits with two VA sites and phone interviews with nine other VA site to better describe facilitators and barriers to implementation of this new practice and identified factors influencing high rates of LST documentation completion. Researchers who conducted the interviews analyzed interview data through applying the Consolidated Framework for Implementation Research. From these efforts, we identified best practices used, including the importance of a formally appointed implementation leader, gaining leadership support, and engaging staff through education. We will share these recommendations with VA sites across the healthcare system to improve LST documentation.

